# Synthesis of Composition-Tunable Ag-Cu Bimetallic Nanoparticles Through Plasma-Driven Solution Electrolysis

**DOI:** 10.3390/nano14211758

**Published:** 2024-10-31

**Authors:** Chi Xu, Himashi P. Andaraarachchi, Uwe R. Kortshagen

**Affiliations:** Department of Mechanical Engineering, University of Minnesota, 111 Church Street SE, Minneapolis, MN 55455, USA; xu000428@umn.edu (C.X.); handaraa@umn.edu (H.P.A.)

**Keywords:** metal nanoparticles, bimetallic, plasma–liquid interface, immiscible metals, Ag-Cu

## Abstract

Bimetallic nanomaterials have shown great potential across various fields of application. However, the synthesis of many bimetallic particles can be challenging due to the immiscibility of their constituent metals. In this study, we present a synthetic strategy to produce compositionally tunable silver–copper (Ag-Cu) bimetallic nanoparticles using plasma-driven liquid surface chemistry. By using a low-pressure nonthermal radiofrequency (RF) plasma that interacts with an Ag-Cu precursor solution at varying electrode distances, we identified that the reduction of Ag and Cu salts is governed by two “orthogonal” parameters. The reduction of Cu^2+^ is primarily influenced by plasma electrons, whereas UV photons play a key role in the reduction of Ag^+^. Consequently, by adjusting the electrode distance and the precursor ratios in the plasma–liquid system, we could control the composition of Ag-Cu bimetallic nanoparticles over a wide range.

## 1. Introduction

Bimetallic nanoparticles (NPs) exhibit excellent optical, catalytic, and electronic properties that differ from those of their individual component metals [1,2,3,4]. Accordingly, there has been significant interest in developing new synthesis methods for these NPs to explore their applications as catalysts [5,6,7,8], in sensors [9,10], as antimicrobial agents [11,12,13], and as substrates for surface-enhanced Raman scattering [14,15]. The physicochemical properties of bimetallic NPs can be significantly influenced by several factors, such as the chemical nature of the constituent metals, particle size, and the nanoscale arrangement of the two metals, e.g., whether homogenous alloys or core–shell or Janus NPs are formed [1,2,3,16,17].

Many bimetallic systems exhibit wide miscibility gaps in their phase diagrams due to their positive heat of mixing. Thus, most conventional methods, such as co-reduction, seed-mediated growth, and thermal decomposition, yield core–shell NPs or phase-segregated heterostructures rather than homogeneous alloys [1,2,3]. However, it has been shown that nonequilibrium methods, such as spark discharge plasmas [18], pulsed laser ablation [19], and the application of rapid current pulses [20], can lead to the successful homogenous mixing of otherwise immiscible metal systems.

As a nonequilibrium method, plasma-driven solution electrolysis (PDSE) has become an emerging strategy for the synthesis of NPs [21,22,23,24,25,26,27]. Nonthermal plasmas excited by AC (alternating current), DC (direct current), or RF electric fields, generate a highly reactive environment including ions, electrons, radicals, and ultraviolet (UV) photons with a distribution of energies [28,29,30]. When the plasma treats an electrolytic solution containing metallic cations (for example, Ag^+^), these plasma-generated species penetrate the plasma–liquid interface. More reactive species including H·, OH·, O_2_^−^, and solvated electrons (e^−^) are produced by solvation of plasma-activated species. These reducing agents reduce the metallic cations to form neutral metal species (Ag^0^). These neutral atoms then undergo nucleation and coalescence, producing larger clusters, leading to the formation of nanoparticles. Without further requiring chemicals as reducing agents, plasmas generate a variety of reducing species on short timescales on the order of μs to prevent NP agglomeration, while the species’ densities can be controlled via plasma operating parameters such as power, electrode distance, pulsing frequency, and duty cycle. The fast, controllable reactions make PDSE a greener and surfactant-free method for nanomaterial synthesis. Compared to the conventional electrochemical synthesis of NPs, the PDSE method demonstrated the advantages of a good Faradaic Efficiency and a high yield with a short treatment time [31,32,33]. In our recent work, we demonstrated the synthesis of silver nanoparticles using a PDSE reactor which featured fast synthesis of sub-10 nm Ag NPs with a residence time as short as a few seconds. The conversion of precursor to NPs can be as high as 70%, while the size of the NPs can be tuned by operating parameters including plasma power, residence time, and plasma pulsing [34,35].

However, the formation of bimetallic nanoparticles in PDSE still remains as a relatively unexplored area. Recently, Chen et al. demonstrated that plasma-driven solution electrolysis (PDSE) can be used to synthesize bimetallic Ag-Pt alloy NPs from typically immiscible metal systems, employing a pulsed plasma that interacts with a precursor solution [36]. But the good control of nanoparticle composition still remains elusive.

Here, we present a synthetic strategy for the formation of composition-tunable bimetallic copper (Cu)–silver (Ag) NPs using low-pressure PDSE. Ag-Cu bimetallic NPs have been reported to have higher electrical conductivity, better oxidation resistance, and catalytic performance than their constituent metals [37,38]. Previous reports have demonstrated the synthesis of Ag-Cu bimetallic NPs using microwave irradiation, thermal decomposition, and chemical reduction; however, these methods require either adding reducing agents or a long treatment time lasting for hours [39,40,41]. Copper and silver are immiscible at almost all composition ratios at room temperature, forming a two-phase mixture. Accordingly, phase-segregated heterostructures have been found in conventional synthetic methods [42,43,44,45].

In this work, we use the Ag-Cu system to demonstrate improved compositional control through nonthermal plasma PDSE. We employed a low-pressure radiofrequency (RF) plasma that interacts with a liquid droplet of a precursor solution containing Ag^+^ and Cu^2+^ salts [34,35]. We recently demonstrated that both plasma electrons and UV photons contribute to the reduction of metal salts in PDSE, with their respective densities decreasing with different rates axially with increasing distance from the electrode [34,35]. Taking advantage of different active species at the plasma–liquid interface, we demonstrate that composition-tunable Ag-Cu bimetallic NPs can be synthesized by tuning the electrode distance and precursor concentrations.

## 2. Materials and Methods

### 2.1. Ag-Cu Bimetallic Nanoparticle Synthesis

**Figure 1:**
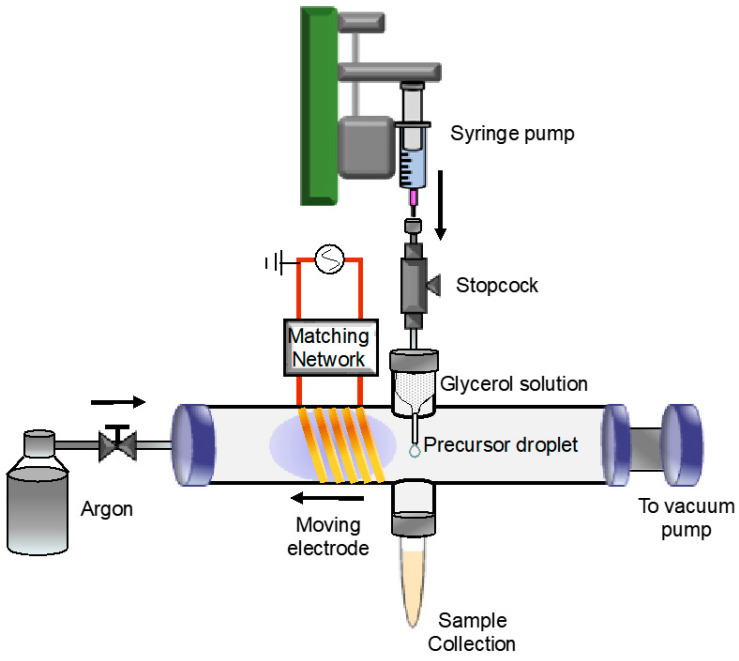
Schematic of plasma–liquid electrolysis reactor for the synthesis of Ag-Cu bimetallic NPs in glycerol. An inductive discharge was generated using an RF power supply that is connected to a five-turn copper coil via a matching network. A mixture of Ag^+^ and Cu^2+^ precursor solution was delivered through a syringe pump that connected to a quartz pipette with a 1 mm tip. The precursor-glycerol droplets, which formed continuously, were directly exposed to the plasma and held by surface tension until they detached from the pipette and collected in a culture tube.

Ag-Cu bimetallic NPs were synthesized and collected in a nonthermal radio RF inductively coupled plasma reactor, shown in Figure 1, which is similar to the method used in a previously published report [34,35]. The discharge gas, argon (Ar) (Airgas, UPC 300, 99.9993%), flowed through a 2.54 cm outer diameter (OD) and 2.20 cm inner diameter (ID) quartz tube at a flow rate of 80 sccm (standard cubic centimeters per minute). The system operated at a total pressure of 5 Torr. Plasma was generated by the application of RF power at 13.56 MHz from a power generator through a five-turn copper induction coil coupled with a matching network (HFT1500, Vectronics, Starkville, MS, USA). The RF power was generated using a B & K Precision 4063 arbitrary function generator (B & K Precision Corporation, Yorba Linda, CA, USA) producing a driving signal with a peak-to-peak voltage ranging from 300 to 620 mV, which was fed into an E&I A300 broadband power amplifier with a gain of 56 dB (Electronics & Innovation, Rochester, NY, USA), generating a nominal plasma power of 80 W.

The precursor solutions were prepared by mixing appropriate amounts of silver nitrate (ACS reagent, ≥99.0%, Sigma-Aldrich, St. Louis, MO, USA) and copper (II) trifluoromethanesulfonate (≥98%, Sigma-Aldrich, St. Louis, MO, USA) in glycerol (ACS reagent, ≥99.5%, Sigma-Aldrich, St. Louis, MO, USA) to achieve the desired Ag:Cu concentrations. The precursor solutions were sonicated for 1 h to enhance the dispersibility of the metal salts. Four different molar ratios of Ag:Cu concentrations, 1:10, 1:100, 1:200, and 1:500, were used in this study.

Precursor solutions were injected into the plasma via a syringe pump (NE-300, New Era Pump Systems, East Farmingdale, NY, USA), and liquid droplets were generated which were allowed to hang at a pipette tip. When the surface tension was no longer sufficient to hold the droplets at the pipette tip, they detached and fell into a culture tube for collection and further characterization. The residence time of each droplet at the pipette tip was approximately 3 s.

The default position of the droplet was located on the quartz tube centerline and axially 8 mm away from the end of the induction coil. The induction coil could not be moved any closer to the coil due to the two glass flanges present in the tube (Figure 1). This position was defined as the reference position, which will be referred to as 0 cm distance. The effect of electrode distance was investigated by moving the induction coil away from the droplet to distances of 1 cm and 2 cm from the reference position.

Collected NPs were purified by centrifugation at 14,000 rpm for 1 h, followed by rinsing with ethanol thrice. The isolated final product was dispersed in ethanol for further characterization.

### 2.2. Transmission Electron Microscopy (TEM)

The purified products were drop-cast on thin carbon-coated copper and molybdenum grids, and dried in a dark fume hood. High-angle annular bright-field scanning transmission microscopy (BF-STEM) images were collected using Thermo Scientific Talos F200X scanning electron microscope equipped with a Super-X energy dispersive X-ray (EDX) detector (Thermo Scientific, Waltham, MA, USA) operating at an accelerating voltage of 200 kV. Spatially resolved STEM EDX maps were collected with an acquisition time of 10 min, and a dwell time of 100 µs/pixel. Each TEM grid was randomly sampled at more than five locations throughout the entire grid area to ensure that the NP composition was uniform. The K-edges of Ag and Cu were background-subtracted and integrated to produce the net intensity elemental maps and line scans.

## 3. Results and Discussion

### 3.1. Effects of Precursor Concentration Ratios

Co-reduction has been suggested as an effective method for achieving homogeneous mixing of two immiscible metals [16]. The key to successful co-reduction is balancing the reduction rates of the two precursors to ensure they react on similar timescales [16]. Given that the reduction potential of Cu^2+^ (0.34 V) is lower than that of Ag^+^ (0.8 V), indicating that Ag^+^ is more easily reduced than Cu^2+^, we selected a higher initial concentration of the Cu precursor compared to the Ag precursor to maintain comparable reduction rates.

We first explored the effects of different concentrations of Ag on the final composition of the products at a reference electrode distance of 0 cm. While the Cu^2+^ concentration was kept constant at 100 mM, the Ag^+^ precursor concentration was varied at 10 mM, 1 mM, 0.5 mM, and 0.2 mM to obtain Ag:Cu precursors with molar concentration ratios of 1:10, 1:100, 1:200, and 1:500.

**Figure 2:**
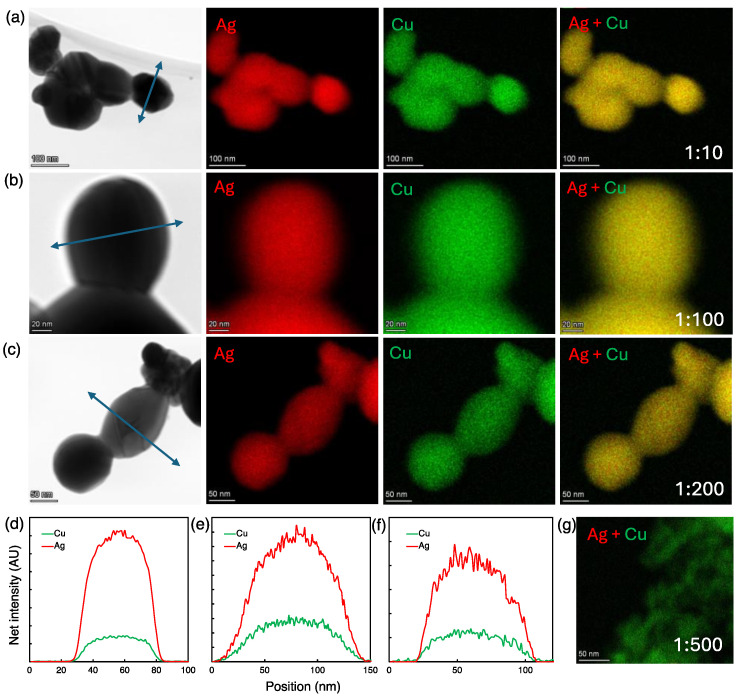
(**a**–**c**) BF-STEM images, Ag EDX elemental maps, Cu EDX elemental maps, and overlay of Ag and Cu EDX elemental maps of Ag-Cu NPs produced from 1:10, 1:100, and 1:200 Ag:Cu precursor ratios at 0 cm electrode distance, respectively. (**d**–**f**) EDS line scan profiles of homogeneously mixed Ag-Cu bimetallic NPs at 1:10, 1:100, and 1:200 Ag:Cu precursor ratios, respectively. (**g**) Ag-Cu overlapped EDS elemental map of Cu NPs formed from 1:500 Ag:Cu precursor ratio at 0 cm electrode distance.

Figure 2 shows the BF-STEM images, Ag and Cu net intensity EDX elemental maps, overlapped Ag-Cu EDX elemental maps, and corresponding line scan profiles at the electrode reference position at 0 cm for varying Ag:Cu concentration ratios. As shown in Figure 2a, at a 1:10 Ag: Cu concentration ratio, the Ag elemental map shows a uniform distribution of Ag throughout the NPs, suggesting that Ag atoms are well dispersed within the particle. Similarly, the Cu elemental map also exhibits a uniform distribution complementing the Ag map, which suggests a homogeneous mixing of both elements. The overlay of the Ag-Cu mixed elemental map further demonstrates the co-localization of Ag and Cu signals with no visible segregation of the two metals with atomic concentrations of 80% Ag and 20% Cu. While these observations do not conclusively confirm the formation of an alloy vs. a microscopic two-phase mixture, Ag and Cu appear more homogeneously mixed than in other literature reports on Ag-Cu NPs. We refer to these particles as “homogenously mixed”.

We observed similar patterns with the 1:100 and 1:200 Ag:Cu concentration ratios where the Ag and Cu elemental maps and overlapped Ag-Cu maps reveal a uniform distribution of Ag and Cu atoms throughout the particles. This indicates a homogenous mixing of Ag and Cu with a composition of an atomic % of about 60% Ag and 40% Cu at both concentration ratios (Figure 2b,c).

Line scans were conducted across different sections of the Ag-Cu bimetallic NPs. Combined line scan profiles, shown in Figure 2d–f for 1:10, 1:100, and 1:200 Ag:Cu ratios, respectively, show uniform distributions of Ag and Cu signals along the scan lines for all concentration ratios, suggesting the formation of homogeneously mixed Ag-Cu NPs at a 0 cm electrode distance.

However, this trend changed at a 1:500 Ag:Cu ratio, as illustrated in Figure 2e. The overlay of Ag-Cu elemental maps shows the formation of Cu NPs without any indication of Ag NP production. This suggests that at Ag:Cu ratios larger than 1:200, Cu^2+^ reduction will become predominant over Ag^+^ reduction leading to the preferential nucleation and growth of Cu NPs rather than the formation of bimetallic or phase-segregated NPs.

### 3.2. Effects of Plasma Electrode Distance

In previous work, we demonstrated that VUV photon-induced dissociation of glycerol is the main factor in the Ag^+^ reduction in our plasma–liquid droplet system [34] and that electron-driven reduction plays a minor role. Here, we hypothesized that the reduction of Cu^2+^ should be much more sensitive to the flux of plasma electrons because it involves two reaction steps. Furthermore, in previous work we found a rapid axial decay of the electron density, observed with double Langmuir probe measurements, but simulations predict a much slower axial decay of VUV fluxes in our reactor configuration [33]. Hence, we hypothesized that the electrode distance should have a significantly stronger impact on the reduction of Cu^2+^ than Ag^+^.

**Figure 3:**
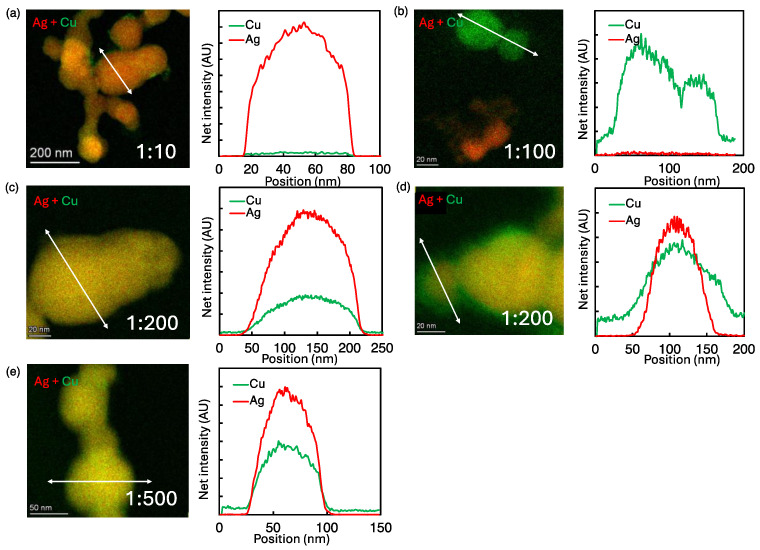
Overlay of Ag-Cu EDS elemental maps and line scan profiles of resultant NPs at 1 cm electrode distance. (**a**) Ag NPs at 1:10 precursor ratio (**b**) phase-segregated Ag and Cu NPs at 1:100 precursor ratio (**c**,**d**) homogeneously mixed Ag-Cu NPs and Ag-Cu core–shell NPs at 1:200 and (**e**) homogeneously mixed Ag-Cu NPs at 1:500 precursor ratio.

To test this hypothesis, we examined the effect of electrode distance by moving the induction coil 1 cm away from the droplet at the same concentration ratios. This resulted in noticeably different observations, as illustrated by Figure 3, which shows the BF-STEM images, overlays of Ag-Cu EDX elemental maps, and the corresponding line scan profiles of Ag-Cu bimetallic NPs at the 1 cm electrode distance.

The overlapped Ag-Cu elemental map of 1:10 Ag:Cu (Figure 3a) primarily shows Ag NPs, with some clusters of Cu NPs also present. This agrees with the combined line scan profile that predominantly exhibits Ag signal with minimal Cu signal throughout the NP. As the Ag:Cu concentration ratio increased to 1:100, clear phase segregation of Ag and Cu NPs was observed, as depicted in Figure 3b, which shows the overlay of the Ag-Cu elemental maps and the line scan profile. This indicates that, despite Cu^2+^ reduction occurring, co-reduction is still not favorable under these conditions.

As the Ag:Cu concentration ratio increased to 1:200, we observed two distinct structures: homogeneously mixed NPs with an even distribution of Ag and Cu atoms, as shown in Figure 3c, and nonhomogenous particles, some of which appeared to be Ag-Cu core–shell NPs, as shown in Figure 3d. At the highest Ag:Cu concentration ratio of 1:500, co-reduction was predominant, resulting in the formation of homogeneously mixed Ag-Cu NPs (Figure 3e). These results clearly indicate that plasma electron density and precursor concentration significantly impact Cu^2+^ reduction, which in turn affects the rate of co-reduction and the composition of Ag-Cu bimetallic NPs.

Our results are consistent with our hypothesis that Cu^2+^ reduction might be mainly influenced by plasma electrons, and not be dominated by VUV photons as in the case of Ag^+^. To further test the hypothesis that Cu^2+^ reduction is electron-dominated, we performed an experiment by moving the induction coil away by a larger distance of 2 cm for two concentrations of 1:10 and 1:500. Due to the much more rapid axial decay of the electron density compared to VUV fluxes, we expected a significant drop in Cu^2+^ reduction.

**Figure 4:**
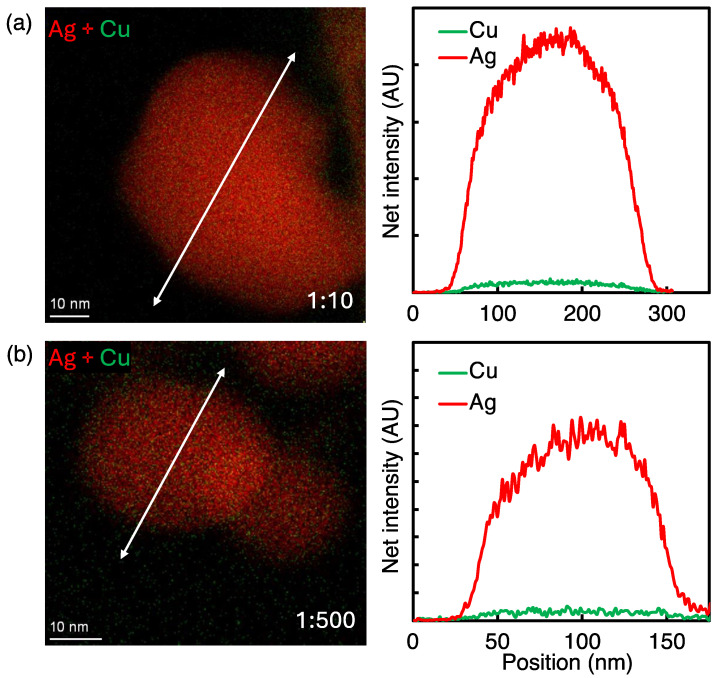
Overlay of Ag-Cu EDS elemental maps and line scan profiles of resultant NPs at 2 cm electrode distance. (**a**) Ag NPs at 1:10 precursor ratio; (**b**) Ag NPs at 1:500 Ag:Cu precursor ratio.

Figure 4 shows Ag-Cu overlay elemental maps and the corresponding line scan profiles for 1:10 and 1:500 Ag:Cu concentrations at the 2 cm electrode distance. Both mixed elemental maps, along with line scan profiles, as shown in Figure 4a,b, clearly show the formation of almost pure Ag NPs, indicating Ag^+^ reduction is dominant under these conditions. These findings further corroborate that the electron flux, as controlled by electrode distance, is a critical parameter for the Cu^2+^ reduction.

### 3.3. Mechanisms of Tuning NP Composition in PDSE

**Figure 5:**
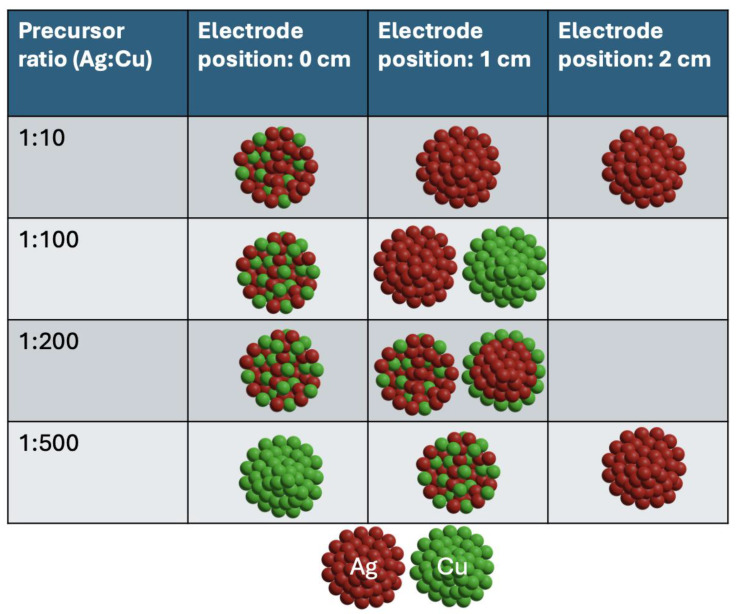
Schematic summary of Ag-Cu bimetallic NPs produced by varying electrode distance and Ag:Cu precursor ratio. Ag NPs denoted by red spheres and Cu NPs denoted by green spheres.

Figure 5 summarizes our findings for the three electrode distances of 0 cm, 1 cm, and 2 cm with varying Ag:Cu precursor concentration ratios. At the closest distance (0 cm), where the electron density is the highest, co-reduction of Ag^+^ and Cu^2+^ leads to the formation of homogeneously mixed Ag-Cu bimetallic NPs at most concentration ratios. As the electron flux drops exponentially with the axial distance, at 1 cm, the rate of Cu^2+^ reduction varies with the precursor concentration ratio, resulting in phase-segregated NPs, core–shell Ag-Cu NPs, and homogeneously mixed Ag-Cu NPs. At the farthest electrode distance of 2 cm, the reduction of Ag^+^ prevails, resulting in the formation of Ag NPs.

The proposed mechanisms are as follows: when the plasma is in direct contact with the liquid, various plasma-generated reactive species penetrate the liquid surface. The plasma-generated electrons become solvated in the liquid phase, while energetic UV photons lead to the photodissociation of glycerol, forming H· radicals [46]. Both e^−^ and H· are important reducing agents for metallic precursor ions, for example, via Ag^+^ + e^−^ → Ag^0^ and Ag^+^ + H· → Ag^0^ [47,48]. For Ag NPs, in our previous studies in this reactor [34,35], we demonstrated that the electron fluxes toward the liquid drop much more drastically than the UV fluxes, and that the Ag NP yield is not sensitive to the electrode distance. In comparison, for the reduction of Cu^2+^, the reaction rate constant *k* of Cu^2+^ reduced by solvated electrons is ~10^10^ M^−1^s^−1^, which is about three orders of magnitude higher than that for other species, including H radicals (*k*~10^7^ M^−1^s^−1^) [49]. Hence, we proposed that the reduction of Cu^2+^ is electron-dominated, which is supported by our above results, as summarized in Figure 5.

**Figure 6:**
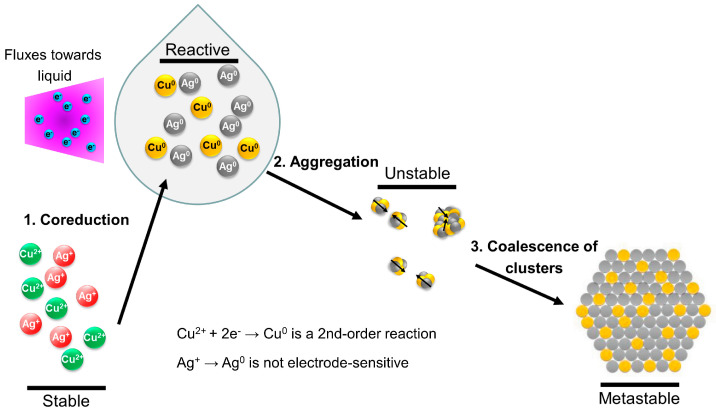
Illustration of the reduction mechanisms for Ag-Cu bimetallic mixing structures. While plasma provides tunable reactive species fluxes toward the liquid, another key parameter to control the reduction rate is the precursor concentration.

As illustrated in Figure 6, in this bimetallic NP system, before the plasma treatment, two metallic precursors are dissolved in the solvent, then dissociate to anions and metallic cations (Ag^+^ and Cu^2+^). These cations are then reduced to neutral atoms, followed by aggregation to clusters and resulting in a mixed configuration. The reduction rates of Ag^+^ and Cu^2+^ may not be comparable, because of their different charges and reduction potentials. Therefore, to maintain the co-reduction, electrode position and precursor concentration ratios are adjusted to control the concentrations of reactive species.

Our results highlight that the composition of Ag-Cu NPs can be controlled, within bounds, by the “orthogonal” parameters of concentration ratio and droplet distance from the plasma: Cu^2+^ reduction is primarily electron-dominated and Ag^+^ reduction is UV photon-dominated. These two plasma-generated species, with different spatial distributions, make the electrode distance an important parameter for tuning the reactive species fluxes. At the same time, the tradeoff of Ag^+^ and Cu^2+^ reduction can be achieved by changing precursor concentration ratios. Thus, control over NP composition can be achieved by optimizing electrode distance and precursor ratios, leveraging the distinct roles of plasma electrons and UV photons in the reduction process.

## 4. Conclusions

In conclusion, this study illustrates that both electrode distance and precursor concentration ratios play crucial roles in determining the composition and structure of Ag-Cu bimetallic NPs in a PDSE system. When the plasma is closest (0 cm), the high electron density enables effective co-reduction of Ag^+^ and Cu^2+^, leading to uniformly mixed Ag-Cu NPs for all precursor ratios, with the exception of the lowest Ag concentration, where only Cu NPs are produced. At the medium electrode distance (1 cm), varying precursor ratios affect the reduction rate of Cu^2+^, resulting in phase-segregated, core–shell, or homogeneously mixed NPs. At the largest distance (2 cm), Ag^+^ reduction prevails, resulting in exclusively Ag NPs. Our findings also reveal that the reduction of Cu^2+^ is mainly driven by plasma electrons, whereas Ag^+^ reduction is dominated by UV photons. Accordingly, controlling NP composition can be achieved by adjusting electrode distance and precursor ratios, taking advantage of the different roles played by plasma electrons and UV photons in the reduction processes. We propose that this scheme may also apply to other bimetallic systems where the reduction of the constituent metal ions is governed by different reduction mechanisms.

## Data Availability

The original contributions presented in this study are included in the article, further inquiries can be directed to the corresponding author.

## References

[1] Idris D.S., Roy A. (2023). Synthesis of Bimetallic Nanoparticles and Applications—An Updated Review. Crystals.

[2] Dlamini N.G., Basson A.K., Pullabhotla V.S.R. (2023). Synthesis and Characterization of Various Bimetallic Nanoparticles and Their Application. Appl. Nano.

[3] Loza K., Heggen M., Epple M. (2020). Synthesis, Structure, Properties, and Applications of Bimetallic Nanoparticles of Noble Metals. Adv. Funct. Mater..

[4] Larrañaga-Tapia M., Betancourt-Tovar B., Videa M., Antunes-Ricardo M., Cholula-Díaz J.L. (2023). Green synthesis trends and potential applications of bimetallic nanoparticles towards the sustainable development goals 2030. Nanoscale Adv..

[5] Liu L., Corma A. (2023). Bimetallic Sites for Catalysis: From Binuclear Metal Sites to Bimetallic Nanoclusters and Nanoparticles. Chem. Rev..

[6] Liu L., Corma A. (2018). Metal Catalysts for Heterogeneous Catalysis: From Single Atoms to Nanoclusters and Nanoparticles. Chem. Rev..

[7] Wang C., Wang K., Feng Y., Li C., Zhou X., Gan L., Feng Y., Zhou H., Zhang B., Qu X. (2021). Co and Pt Dual-Single-Atoms with Oxygen-Coordinated Co-O-Pt Dimer Sites for Ultrahigh Photocatalytic Hydrogen Evolution Efficiency. Adv. Mater..

[8] Hao B., Gunaratna M.J., Zhang M., Weerasekara S., Seiwald S.N., Nguyen V.T., Meier A., Hua D.H. (2016). Chiral-Substituted Poly-N-Vinylpyrrolidinones and Bimetallic Nanoclusters in Catalytic Asymmetric Oxidation Reactions. J. Am. Chem. Soc..

[9] Liu N., Tang M.L., Hentschel M., Giessen H., Alivisatos A.P. (2011). Nanoantenna-Enhanced Gas Sensing in a Single Tailored Nanofocus. Nat. Mater..

[10] Huan K., Li Y., Deng D., Wang H., Wang D., Li M., Luo L. (2022). Composite-Controlled Electrospinning of CuSn Bimetallic Nanoparticles/Carbon Nanofibers for Electrochemical Glucose Sensor. Appl. Surf. Sci..

[11] Arora N., Thangavelu K., Karanikolos G.N. (2020). Bimetallic Nanoparticles for Antimicrobial Applications. Front. Chem..

[12] Perdikaki A., Galeou A., Pilatos G., Karatasios I., Kanellopoulos N.K., Prombona A., Karanikolos G.N. (2016). Ag and Cu Monometallic and Ag/Cu Bimetallic Nanoparticle-Graphene Composites with Enhanced Antibacterial Performance. ACS Appl. Mater. Interfaces.

[13] Paszkiewicz M., Golabiewska A., Rajski L., Kowal E., Sajdak A., Zaleska-Medynska A. (2016). Synthesis and Characterization of Monometallic (Ag, Cu) and Bimetallic Ag-Cu Particles for Antibacterial and Antifungal Applications. J. Nanomater..

[14] Ma J., Liu X., Wang R., Zhang J., Jiang P., Wang Y., Tu G. (2020). Bimetallic Core-Shell Nanostars with Tunable Surface Plasmon Resonance for Surface-Enhanced Raman Scattering. ACS Appl. Nano Mater..

[15] Awiaz G., Lin J., Wu A. (2023). Recent Advances of Au@Ag Core–Shell SERS-based Biosensors. Exploration.

[16] Gilroy K.D., Ruditskiy A., Peng H.-C., Qin D., Xia Y. (2016). Bimetallic Nanocrystals: Syntheses, Properties, and Applications. Chem. Rev..

[17] Wang D., Li Y. (2011). Bimetallic nanocrystals: Liquid-phase Synthesis and Catalytic Applications. Adv. Mater..

[18] Tabrizi N.S., Xu Q., van der Pers N.M., Schmidt-Ott A. (2010). Generation of Mixed Metallic Nanoparticles from Immiscible Metals by Spark Discharge. J. Nanopart. Res..

[19] Kane K.A., Reber A.C., Khanna S.N., Bertino M.F. (2018). Laser Synthesized Nanoparticle Alloys of Metals with Bulk Miscibility Gaps. Prog. Nat. Sci. Mater. Int..

[20] Yang C., Ko B.H., Hwang S., Liu Z., Yao Y., Luc W., Cui M., Malkani A.S., Li T., Wang X. (2020). Overcoming Immiscibility toward Bimetallic Catalyst Library. Sci. Adv..

[21] Boruah P.J., Kalita P., Bailung H. (2022). In-Liquid Plasma: A Novel Tool for Nanofabrication. Plasma Science and Technology.

[22] Chen Q., Li J., Li Y. (2015). A Review of Plasma–Liquid Interactions for Nanomaterial Synthesis. J. Phys. D Appl. Phys..

[23] Bratescu M.A., Cho S.-P., Takai O., Saito N. (2011). Size-Controlled Gold Nanoparticles Synthesized in Solution Plasma. J. Phys. Chem. C.

[24] Kondeti V.S.S.K., Gangal U., Yatom S., Bruggeman P.J. (2017). Ag+ Reduction and Silver Nanoparticle Synthesis at the Plasma–Liquid Interface by an RF Driven Atmospheric Pressure Plasma Jet: Mechanisms and the Effect of Surfactant. J. Vac. Sci. Technol. A.

[25] Mashimo T., Tamura S., Yamamoto K., Kelgenbaeva Z., Ma W., Tokuda M., Koinuma M., Isobe H., Yoshiasa A. (2020). Synthesis of Pd-Ru Solid-Solution Nanoparticles by Pulsed Plasma in Liquid Method. RSC Adv..

[26] Meiss S.A., Rohnke M., Kienle L., Zein El Abedin S., Endres F., Janek J. (2007). Employing Plasmas as Gaseous Electrodes at the Free Surface of Ionic Liquids: Deposition of Nanocrystalline Silver Particles. ChemPhysChem.

[27] Tokushige M., Yamanaka T., Matsuura A., Nishikiori T., Ito Y. (2009). Synthesis of Magnetic Nanoparticles (Fe and FePt) by Plasma-Induced Cathodic Discharge Electrolysis. IEEE Trans. Plasma Sci..

[28] Raisanen A.L., Mueller C.M., Chaudhuri S., Schatz G.C., Kushner M.J. (2022). A reaction mechanism for plasma electrolysis of AgNO_3_ forming silver nanoclusters and nanoparticles. J. Appl. Phys..

[29] Bruggeman P.J., Frontiera R.R., Kortshagen U.R., Kushner M.J., Linic S., Schatz G.C., Andaraarachchi H., Exarhos S., Jones L.O., Mueller C.M. (2021). Plasma-driven solution electrolysis. J. Appl. Phys..

[30] Pang Y., Li H., Hua Y., Zhang X., Di L. (2024). Rapid Synthesis of Noble Metal Colloids by Plasma–Liquid Interactions. Materials.

[31] Bulusu R.K.M., Yatom S., Patterson C.W., Wandell R.J., Locke B.R. (2022). Effects of frequency and pulse width on electron density, hydrogen peroxide generation, and perfluorooctanoic acid mineralization in a nanosecond pulsed discharge gas-liquid plasma reactor. J. Vac. Sci. Technol. A.

[32] Ghosh S., Hawtof R., Rumbach P., Go D.B., Akolkar R., Sankaran R.M. (2017). Quantitative Study of Electrochemical Reduction of Ag^+^ to Ag Nanoparticles in Aqueous Solutions by a Plasma Cathode. J. Electrochem. Soc..

[33] Maguire P., Rutherford D., Macias-Montero M., Mahony C., Kelsey C., Tweedie M., Perez-Martin F., McQuaid H., Diver D., Mariotti D. (2017). Continuous In-Flight Synthesis for On-Demand Delivery of Ligand-Free Colloidal Gold Nanoparticles. Nano Lett..

[34] Xu C., Andaraarachchi H.P., Xiong Z., Eslamisaray M.A., Kushner M.J., Kortshagen U.R. (2023). Size-Tunable Silver Nanoparticle Synthesis in Glycerol Driven by a Low-Pressure Nonthermal Plasma. J. Phys. D Appl. Phys..

[35] Xu C., Chaudhuri S., Held J., Andaraarachchi H.P., Schatz G.C., Kortshagen U.R. (2023). Silver Nanoparticle Synthesis in Glycerol by Low-Pressure Plasma-Driven Electrolysis: The Roles of Free Electrons and Photons. J. Phys. Chem. Lett..

[36] Chen H.T., Lee D., Linic S. (2023). Formation of Mixed Bimetallic Nanoparticles of Immiscible Metals through Plasma-Induced Reduction of Precursors in Solutions: A Case Study of Ag-Pt Alloy Nanoparticles. Chem. Mater..

[37] Hsieh J., Hung S. (2016). The Effect of Cu:Ag Atomic Ratio on the Properties of Sputtered Cu-Ag Alloy Thin Films. Materials.

[38] Lee C., Kim N.R., Koo J., Lee Y.J., Lee H.M. (2015). Cu-Ag core-shell nanoparticles with enhanced oxidation stability for printed electronics. Nanotechnology.

[39] Chen Z., Mochizuki D., Maitani M.M., Wada Y. (2013). Facile synthesis of bimetallic Cu-Ag nanoparticles under microwave irradiation and their oxidation resistance. Nanotechnology.

[40] Kim N.R., Shin K., Jung I., Shim M., Lee H.M. (2014). Ag–Cu Bimetallic Nanoparticles with Enhanced Resistance to Oxidation: A Combined Experimental and Theoretical Study. J. Phys. Chem. C.

[41] Rout L., Kumar A., Dhaka R.S., Dash P. (2016). Bimetallic Ag–Cu alloy nanoparticles as a highly active catalyst for the enamination of 1,3-dicarbonyl compounds. RSC Adv..

[42] Ferrando R., Jellinek J., Johnston R.L. (2008). Nanoalloys: From Theory to Applications of Alloy Clusters and Nanoparticles. Chem. Rev..

[43] Osowiecki W.T., Ye X., Satish P., Bustillo K.C., Clark E.L., Alivisatos A.P. (2018). Tailoring Morphology of Cu–Ag Nanocrescents and Core–Shell Nanocrystals Guided by a Thermodynamic Model. J. Am. Chem. Soc..

[44] Rapallo A., Rossi G., Ferrando R., Fortunelli A., Curley B.C., Lloyd L.D., Tarbuck G.M., Johnston R.L. (2005). Global Optimization of Bimetallic Cluster Structures. I. Size-Mismatched Ag–Cu, Ag–Ni, and Au–Cu Systems. J. Chem. Phys..

[45] Wu W., Lei M., Yang S., Zhou L., Liu L., Xiao X., Jiang C., Roy V.A.L. (2015). A One-Pot Route to the Synthesis of Alloyed Cu/Ag Bimetallic Nanoparticles with Different Mass Ratios for Catalytic Reduction of 4-nitrophenol. J. Mater. Chem. A.

[46] Bell F., Ruan Q.N., Golan A., Horn P.R., Ahmed M., Leone S.R., Head-Gordon M. (2013). Dissociative photoionization of glycerol and its dimer occurs predominantly via a ternary hydrogen-bridged ion-molecule complex. J. Am. Chem. Soc..

[47] Wu H., Liu Z., Xu L., Wang X., Chen Q., Ostrikov K. (2021). The Ag^+^ Reduction Process in a Plasma Electrochemical System Tuned by the pH Value. J. Electrochem. Soc..

[48] Abid J.P., Wark A.W., Brevet P.F., Girault H.H. (2002). Preparation of silver nanoparticles in solution from a silver salt by laser irradiation. Chem. Commun..

[49] Buxton G.V., Greenstock C.L., Helman W.P., Ross A.B. (1988). Critical Review of rate constants for reactions of hydrated electrons, hydrogen atoms and hydroxyl radicals (·OH/·O^−^) in Aqueous Solution. J. Phys. Chem. Ref. Data.

